# Risk Assessment by a Passenger of an Autonomous Vehicle Among Pedestrians: Relationship Between Subjective and Physiological Measures

**DOI:** 10.3389/fnrgo.2021.682119

**Published:** 2021-11-22

**Authors:** Jeffery Petit, Camilo Charron, Franck Mars

**Affiliations:** ^1^Université de Nantes, Centrale Nantes, CNRS, LS2N, Nantes, France; ^2^Université Rennes 2, Rennes, France

**Keywords:** autonomous driving, passenger perception, risk assessment, skin conductance, driving simulator, Bayesian network

## Abstract

Autonomous navigation becomes complex when it is performed in an environment that lacks road signs and includes a variety of users, including vulnerable pedestrians. This article deals with the perception of collision risk from the viewpoint of a passenger sitting in the driver's seat who has delegated the total control of their vehicle to an autonomous system. The proposed study is based on an experiment that used a fixed-base driving simulator. The study was conducted using a group of 20 volunteer participants. Scenarios were developed to simulate avoidance manoeuvres that involved pedestrians walking at 4.5 kph and an autonomous vehicle that was otherwise driving in a straight line at 30 kph. The main objective was to compare two systems of risk perception: These included subjective risk assessments obtained with an analogue handset provided to the participants and electrodermal activity (EDA) that was measured using skin conductance sensors. The relationship between these two types of measures, which possibly relates to the two systems of risk perception, is not unequivocally described in the literature. This experiment addresses this relationship by manipulating two factors: The time-to-collision (TTC) at the initiation of a pedestrian avoidance manoeuvre and the lateral offset left between a vehicle and a pedestrian. These manipulations of vehicle dynamics made it possible to simulate different safety margins regarding pedestrians during avoidance manoeuvres. The conditional dependencies between the two systems and the manipulated factors were studied using hybrid Bayesian networks. This relationship was inferred by selecting the best Bayesian network structure based on the Bayesian information criterion. The results demonstrate that the reduction of safety margins increases risk perception according to both types of indicators. However, the increase in subjective risk is more pronounced than the physiological response. While the indicators cannot be considered redundant, data modeling suggests that the two risk perception systems are not independent.

## 1. Introduction

Traveling in autonomous vehicles changes the driver's role when they become a passenger after ceding control to an automated system (Reilhac et al., 2016; Kyriakidis et al., 2019). Verberne et al. (2012) suggested that individuals do not change their social rules when interacting with an automated system. Basu et al. (2017) supported this idea by revealing that most drivers prefer a driving style that resembles their own. This consideration is all the more important when traveling in a dense space where different types of vulnerable road users (e.g., pedestrians and cyclists) can circulate freely. Such spaces are beginning to appear in Europe and are known as shared spaces (Hamilton-Baillie, 2008). They are designed to eliminate any segregation between road users (e.g., through a lack of signs and road markings). As a result, the fluidity of mobility in these areas essentially relies on social conventions, especially in crowded situations. One of the objectives of this urban design is to enable drivers to better integrate into multi-user environments by reducing vehicle speeds and improving traffic flow (Hamilton-Baillie, 2008; Kaparias et al., 2012; Moody and Melia, 2014). However, such designs have also introduced a new challenge for autonomous vehicles that must navigate among other users in non-signalled areas (e.g., the problem of crowd navigation as discussed by Bresson et al., 2019). The trajectories followed by the vehicle to navigate within this type of environment must remain acceptable to the users around the vehicle but also to the driver-passenger inside. Many studies currently investigate the communication between the autonomous vehicle and pedestrians through external human-machine interfaces (Faas et al., 2020; Métayer and Coeugnet, 2021), but it is also important to ensure that the passenger does not fear a collision risk (e.g., when the vehicle adopts proactive navigation, Kabtoul et al., 2020). It is therefore essential to study what will determine the acceptability of the vehicle's trajectory relative to other road users.

### 1.1. Vehicle Dynamics and Passenger Risk Perception

Gibson and Crooks (1938) proposed the existence of a dynamic space that the driver perceives as an area in which they can navigate safely. The authors named it the “field of safe travel.” This zone represents an envelope of acceptable trajectories for a vehicle. It depends on the driver's experience, the safety distances they wish to respect and their perception of the size of the car, among other factors. Based on these considerations, Kolekar et al. (2020a,b) proposed the driving risk field to model the importance that a driver ascribes to an obstacle that blocks a straight trajectory. In their work, the authors built upon Näätänen and Summala's theory (Näätänen and Summala, 1976), who defined perceived risk as a function of both the subjective importance given to a hazard and the consequences that this hazard could pose. Kolekar et al. (2020a,b) hypothesized that the subjective importance that is given to a risk is proportional to the driver's reaction at the steering wheel when confronted with an obstacle in their trajectory. Using this perspective, the authors developed a measure proportional to the perceived risk if a hazard's consequences remain unchanged (e.g., collision with the same obstacle). Other researchers have found that the values of time-to-collision (TTC) or time headway when following a vehicle or approaching a slower obstacle highly correlate with a driver's perception of a collision (Vogel, 2003; Chen et al., 2016; Zhao et al., 2020). Researchers have particularly investigated TTC in the literature and have demonstrated that it is directly perceived through retinal expansion (Lee, 1976; Bootsma et al., 1997; Bootsma and Craig, 2003). When approaching an obstacle, an autonomous vehicle must initiate an avoidance manoeuvre to avoid a collision. When the path of the vehicle deviates from the obstacle, measures such as the TTC or time headway are no longer relevant while the vehicle continues to approach. In this case, new metrics must be used to study risk perception. Ferrier-Barbut et al. (2018) revealed the existence of a comfort zone that is perceived by the passenger of an autonomous vehicle when the vehicle is passing close to a pedestrian. This suggests that absolute distance is a factor in the passenger's perception. During an avoidance manoeuvre, this distance corresponds to the lateral distance (which is also referred to as the offset) between the vehicle and the obstacle. In summary, the study of a passenger's risk perception that involves an autonomous vehicle must integrate vehicle-environment dynamics.

### 1.2. The Hypothesis of Two Risk Perception Systems

Slovic et al. (2004) described two risk perception systems that are involved in evaluation and decision-making processes when an individual is faced with a potential hazard. “Risk as analysis,” according to the authors, is a system of risk perception that is based on conscious reasoning and uses formal logic. This method for perceiving risk is a common conception in the scientific literature. It assumes that individuals perceive risk by estimating the product of the probability of a hazard and its consequences. However, this type of risk perception, which is slow and costly in cognitive resources, would not be solicited in the event of an imminent threat. Slovic et al. (2004) suggested the existence of a second system of perception that is predominant in this type of situation, which they named “risk as feelings.” This system comprises a quick and reactive way of perceiving risk and is intuitive in nature as it is based on affects. This duality of risk perception aligns with the vision of Loewenstein et al. (2001), who suggested that decision-making results not only from cognitive processing but also from an instinctive and spontaneous emotional appraisal.

This vision of the dual process of risk perception is part of the broader problem regarding the distinction between cognitive processes that are fast, automatic and unconscious (type 1) and those that are slow, laborious and conscious (type 2) (Evans, 2008; Evans and Stanovich, 2013). Risk as analysis would belong to type 2 processes, which rely on working memory and involve the mental simulation of future possibilities to formulate explicit judgements. In contrast, risk as feeling would belong to type 1 processes, which are autonomous, do not require working memory and underlie implicit information processing. However, as Evans (2008) rightly pointed out, the nature of the distinction between the two types of processes and their mutual relations are not univocal in the literature. This study addresses this issue through the prism of risk perception in a specific context, that of autonomous vehicles navigating shared spaces.

### 1.3. Risk Measurement

Herrero-Fernández et al. (2020) associated the concept of risk as analysis with a subjective assessment (SA) and associated the concept of risk as feelings with an objective evaluation based on an individual's physiological state. These two evaluation systems are complementary. If an SA consists of measuring the self-reflexive part of risk perception, then the physiological variables provide information on the physical manifestations of this same perception. From this perspective, Choi et al. (2019) considered that, at a certain level, risk perception may require the regulation of the autonomic nervous system, particularly by the sympathetic nervous system. The latter ensures that physiological adaptations occur in preparation for an escape or a struggle when a person is confronted with a stressful event. Such adaptations can manifest as increased cardiac and respiratory rhythms and variations in electrodermal activity (EDA). EDA corresponds to electrical variations in the skin that occur in relation to the functioning of the sweat glands, which are under the control of the sympathetic nervous system (Morange-Majoux, 2017). The most studied property of EDA is skin conductance, which is measured in micro-siemens and consists of the superimposition of two distinct parts that are called “tonic” and “phasic,” respectively. The tonic component is associated with a global skin conductance level (SCL). It is relative to an individual and can be recorded when and individual is at rest. This component reveals slow variations, whereas the phasic component generally reveals rapid changes in skin conductance, which are often called “skin conductance responses (SCRs).” Choi et al. (2019) considered that risk perception could lead to substantial changes in EDA; therefore, EDA could be a good indicator of risk perception. SCRs have already been used as indicators of events that cause stress or discomfort in drivers. For example, Distefano et al. (2020) conducted an experiment using a driving simulator that revealed changes in the EDA of their participants as they approached intersections or roundabouts. Daviaux et al. (2020) observed similar effects when participants in their study were confronted with different driving situations (e.g., the insertion of another vehicle into the lane, crossing with another vehicle going in the opposite direction and crossing with a pedestrian). Skin conductance can be measured non-invasively using two electrodes that are placed on the surface of the skin (Fowles et al., 1981; Boucsein, 2012). In a detailed review about EDA, Boucsein (2012, p. 104–109) presented two preferable sites for placing the electrodes: The hand and the foot. However, the Society for Psychophysiological Research (Society for Psychophysiological Research *Ad Hoc* Committee on Electrodermal Measures, 2012) recommended placing the electrodes on the distal phalanges of the index and middle fingers to obtain bipolar recordings.

To investigate risk perception, this study proposes coupling this physiological measurement with a real-time subjective assessment by using an analogue device that can be operated using one hand. A similar method was used by Rossner and Bullinger (2019, 2020a,b). In their study, the participants were asked to assess their levels of comfort while they were on board a simulated autonomous vehicle. The advantage of collecting an online subjective measurement is that it provides access to the dynamics of changes in perceived risk, unlike verbal or written assessments that involve either an interruption in the task or an *a posteriori* evaluation. The device developed for this study resembles the slide potentiometer used by Walker et al. (2019a). In their study, the authors proposed an experiment to validate their measurement device by asking participants (pedestrians) to assess their willingness to cross a road in real-time while observing an approaching vehicle. The authors concluded that such a continuous assessment device could be useful for assessing human interactions with automated vehicles.

### 1.4. Theoretical Hypothesis

As mentioned previously, two factors that are related to vehicle-environment dynamics were manipulated: The value of the TTC at the moment the vehicle initiates an avoidance manoeuvre and the offset distance left between the vehicle and the pedestrian. It was postulated that the two factors would successively influence passengers' perceived risk. First, based on the results from Bootsma and Craig's study (Bootsma and Craig, 2003), it was assumed that perceived risk increases when the TTC at the time the manoeuvre is initiated decreases. Second, it was assumed that the closer the vehicle passes to a pedestrian (that is, the lower the offset distance), the greater the perceived risk becomes. Both TTC and offset were manipulated to investigate the relationship between the two types of risk perception measures (i.e., the subjective assessment and skin conductance response). From a statistical point of view, the objective was to determine whether the independence of the two types of risk perception was probable given the data and the effects of the independent factors. It was assumed that the measures of SA and SCR were continuous random variables. The purpose of this study was to test whether the experimental data would support the independence between those two variables given the levels of the factors being manipulated. Two alternatives were considered:

- $H_{0}$: SA and SCR are independent despite the measures and levels of factors, which means that the two types of risk perception are independent.
- $H_{1}$: SA and SCR are not independent in at least one combination of measures and factors, which means that there is a relationship between the two types of risk perception.

To address these theoretical hypotheses, hybrid Bayesian networks were implemented. This method, which is based on stochastic distributions, aided in discovering the best structure of relationships (i.e., the one that best fits the data) between manipulated factors and dependant measures. Specifically, Bayesian networks were used to determine whether a relationship between the two risk perception systems could exist and be relevant apart from the assumed influence of the independent environmental factors. In other words, this method was used to assess the significance of the relationship between measures from two types of risk perception given the effects of the TTC and the lateral offset.

Finally, a network coefficient analysis was performed to quantify the effects of the manipulated factors. This analysis was conducted to test the extent to which the influence of the factors was confirmed. That is, this analysis was conducted to validate that a lower TTC at the beginning of an avoidance manoeuvre or a lower offset distance between a vehicle and a pedestrian results in a higher level of risk perception.

## 2. Method

### 2.1. Participants

For this experiment, 20 participants (13 men and 7 women) were recruited. They were between 18 and 52 years old (*M* = 27.1, *SD* = 8.9). Eighteen participants had held driving licenses for 9.8 years on average (*SD* = 9.5) and drove ~11,400 km per year (*SD* = 19,100). The two remaining participants did not possess driving licenses, with the assumption that their perception of risk depended on the same processes as ordinary drivers. In addition, having a driver's license was not considered a prerequisite to be a passenger of a fully autonomous vehicle. Preliminary inspection of the EDA recordings and subjective assessments confirmed that the responses of the two unlicensed participants were not distinct from those of the others.

### 2.2. Experimental Design

A within-subject design was used so that participants would experience a series of 32 pedestrian avoidance maneuvers. The order of presentation of the pedestrians was randomized. They were divided into two blocks of ~7 min presented in succession with a short break in between. The autonomous vehicle was programmed to drive at a constant speed of 30 kph on a 20-m-wide street. The vehicle followed a straight trajectory except when it had to avoid pedestrians. In the real world, the speed limit in shared space is generally lower. According to the British Department of Transport (Great Britain and Department of Transport, 2011), a speed of no more than 20 mph (~32 kph) and preferably <15 mph (~24 kph) is desirable. However, some preliminary experiments revealed that at low vehicle speeds, avoidance manoeuvres elicit very little risk perception from participants. This phenomenon may be explained by the relatively limited immersion of the driving simulator and participants' ability to predict the behavior of the autonomous vehicle. Therefore, the speed of 30 kph was chosen to increase the chances of eliciting risk perception from the participants.

**Figure 1:**
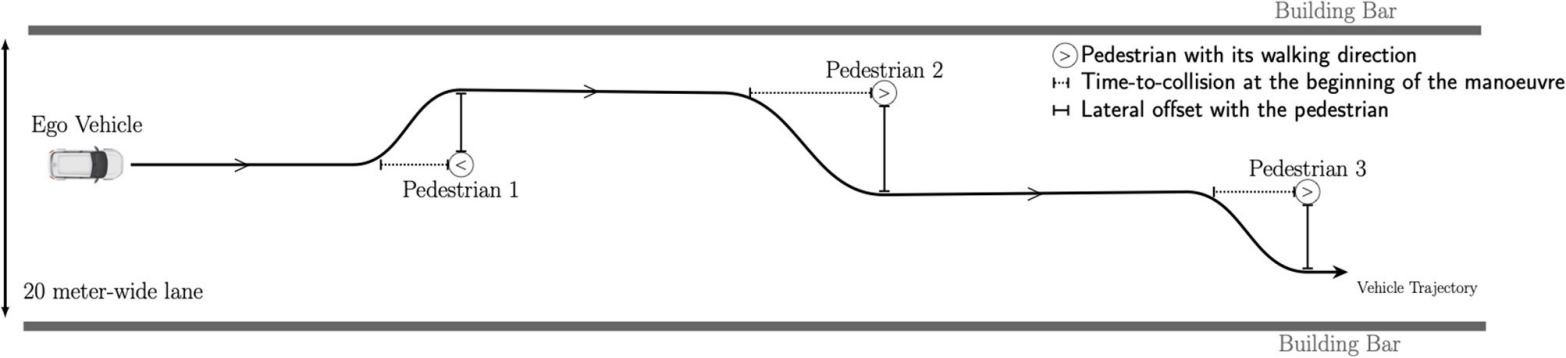
Sample diagram of the vehicle trajectory among pedestrians. The combination of the two factors (TTC and offset) varied between pedestrians according to the experimental plan, which was identical for all the participants. The pedestrians' walking directions and the lateral directions of the avoidance manoeuvres are also represented.

A sample of the vehicle's trajectory is illustrated in Figure 1. After passing a pedestrian, the vehicle did not return to its initial position but maintained its position in the lane until the next pedestrian was encountered. Each pedestrian walked at 4.5 kph and was 25 s apart from the others.

### 2.3. Factors

Two factors acting successively were manipulated (cf. Figure 1).

First, the value of the TTC when the avoidance manoeuvre was initiated was manipulated. In a straight line, the TTC represented the time remaining before the vehicle reached an obstacle. This depended on both the distance and the relative speed between the vehicle and the obstacle. During the experiment, four levels of TTC were tested: 2.0, 2.5, 3.0, and 3.5 s.

Second, the lateral offset distance (simply denoted “the offset”) was manipulated during ongoing avoidance manoeuvres when the vehicle arrived next to a pedestrian. This parameter was introduced to test whether the proximity between the vehicle and the pedestrian affected the participant's perceived risk. Three levels of lateral offset were tested: 0.5, 1.0, and 1.5 m.

It was not possible to combine all the levels of the two factors. Indeed, combining a time-to-collision of 2.0 s and an offset of 1.0 or 1.5 m gave rise to unrealistic vehicle behavior that was caused by the driving simulation software. As a result, only 8 out of the 12 combinations were tested.

**Figure 2:**
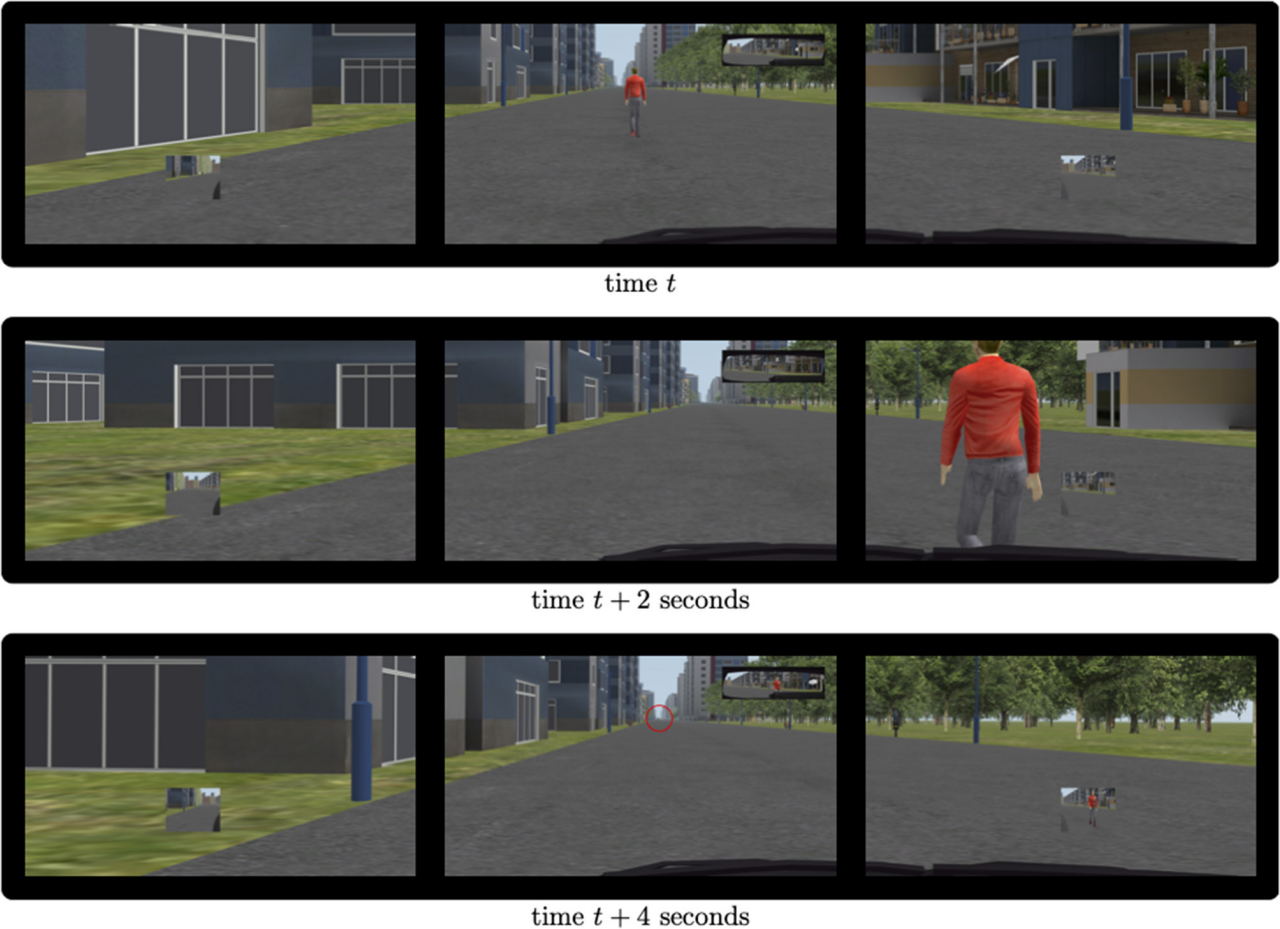
Example of the participant's 120° view during a left avoidance manoeuvre. **(Top)** The approaching phase of a pedestrian walking in the same direction as the vehicle. **(Middle)** The vehicle's position 2 s later when it had started its avoidance manoeuvre. **(Bottom)** The end of the manoeuvre. The avoided pedestrian is visible in the left and center rear-view mirrors. The following pedestrian (circled in red) can hardly be distinguished. This is because each avoidance manoeuvre was separated by 25 s to ensure the independence of the measurements.

Two additional factors were introduced to make the simulations more realistic and unpredictable. Half of the pedestrians walked in the direction in which the vehicle was moving, while the other half walked in the direction opposite the vehicle. In a shared space, there are no rules regarding the direction in which a vehicle should go to avoid other road users. For this reason, the direction of the avoidance manoeuvres varied between left and right. Preliminary statistical analyses, which have not been reported here, demonstrated that these two factors did not affect the results. Finally, the appearance of each pedestrian was arbitrarily chosen from a list of a dozen possibilities (a man or a woman in a t-shirt or a suit, a teenager in shorts, etc.). Figure 2 illustrates what the participants saw during a left avoidance manoeuvre in three screenshots.

### 2.4. Experimental Setup

**Figure 3:**
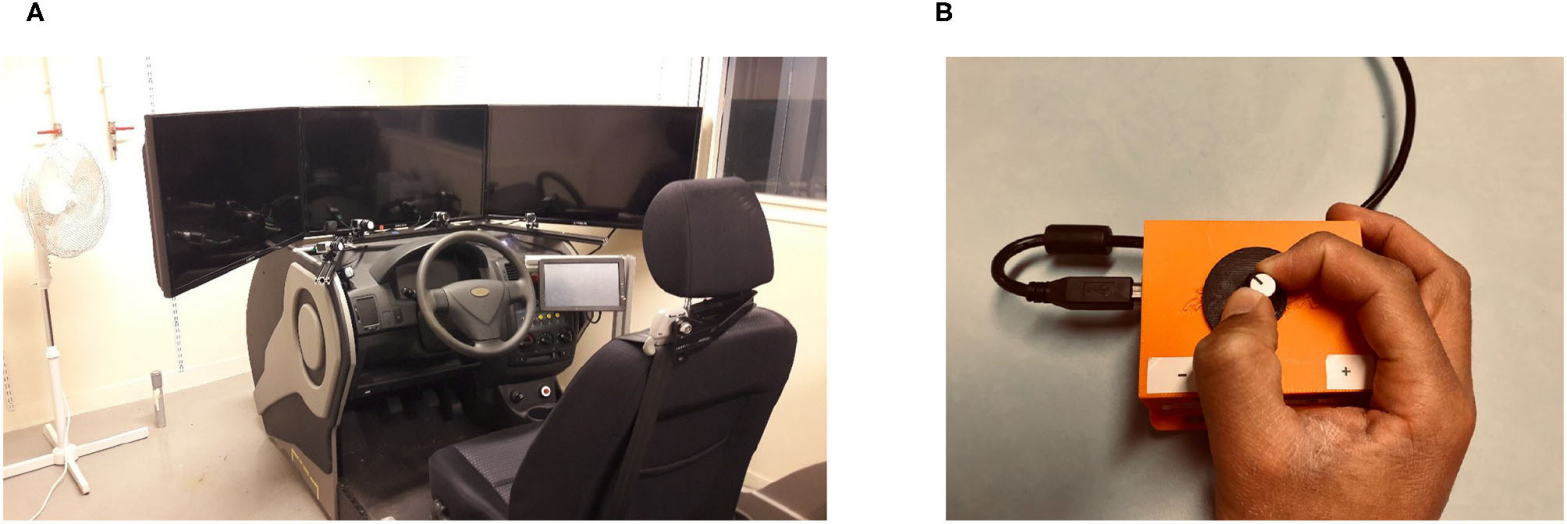
Experimental setup. **(A)** A fixed-base driving simulator. **(B)** Analogue device for subjective risk assessment.

The experiment took place using a fixed-base driving simulator that was run using SCANeR Studio™ software (AVSimulation, France). This driving simulator provides visibility of 120° thanks to three screens (see Figure 3A). During the simulation, the participants were informed that the vehicle was fully automated and that they did not need to use the controls.

In order to record physiological responses during the simulation, the participants' skin conductance was measured according to recommendations of the Society for Psychophysiological Research (Society for Psychophysiological Research *Ad Hoc* Committee on Electrodermal Measures, 2012, p. 6-7). Two electrodes were placed on the distal phalanges of the participants' index and middle fingers on their non-dominant hands. To improve the skin-electrode electrical conductivity and the accuracy of the data collection, the electrodes were covered with isotonic gel. No skin preparation was done before the electrodes were placed. Data recording began at least 5 min after establishing electrode-skin contact to create better electrical contact and stabilize the baseline (that is, the skin conductance level). The data were collected at 625 Hz on a dedicated computer using the software AcqKnowledge 5.0 (BIOPAC Systems, Inc., USA), which was coupled with an acquisition module (16 bits analogue to digital converter; MP160 system, Systems, Inc., USA).

To enable the participants to assess perceived risk throughout the simulation, an analogue device (a potentiometer that was connected to an Arduino^*TM*^ Uno board) was developed for one-handed use, which is illustrated in Figure 3B. The device was designed so that it did not cause visual distraction and could be used without the participants having to look at it. The device was placed on the participants' laps in such a way that they could manipulate it using their dominant hands (i.e., the hand that did not have the conductance measurement electrodes attached to it). Data were collected and linked to the autonomous driving scenarios with a sampling rate of 20 Hz.

### 2.5. Procedure

Participants were asked to fill out a form to provide information about their ages, genders, and driving experience. They were then informed of the purpose and the course of the experiment. At the end of the introductory period, the electrodes were installed according to the manual preferences of the participants. Each participant was then invited to sit in the simulator; at this point, the analogue device was presented and handed over to them.

Each participant was presented with a preliminary scenario that consisted of autonomously driving on a road without pedestrians. Each participant's objective was to optimize the use of the hand-held device by finding a good position for their hands and exploring the rating scale available. Voluntarily, no scale or reference value that was related to risk assessment was provided to the participants. Therefore, they had no prior knowledge of the lowest or highest levels of stimulation that they would encounter. This approach was inspired by Stevens' book about psychophysics (Stevens, 2017, p. 28): This testing method gave participants more freedom without distracting them as it did not require them to do calculations to scale their responses to a certain criterion. The participants were expected to pay attention as much as possible to the driving scene and ignore the assessment device. During this scenario, a horizontal gauge indicating the cursor's position in real-time was displayed on the central monitor. The gauge represented the position of the cursor in the usual way (that is, with the minimum value on the left and the maximum value on the right). The participants were initially invited to adjust their positions in the driver's seat and to familiarize themselves with the assessment device without the researcher's intervention. They were then asked to perform a few exercises: Starting from either the cursor's minimum or maximum position, they had to reach the first third, the median and then two-thirds of the gauge. During this training phase, the participants were asked to close their eyes and reopen them when they thought they had reached the required positions. In this way, they could estimate their errors and readjust their positions if necessary. An error of 5% around the target position was allowed. This phase ended as soon as the participants managed to reach all the required positions and felt confident enough to reach any other position. This training period also made it possible to check the quality of the physiological data collected (EDA) and to adjust the electrodes' placement if necessary.

Afterwards, the participants were instructed to experiment “using the analogue device to assess their risk of collision with pedestrians in real-time when moving in a shared space.”

### 2.6. Calculation of Dependent Variables

#### 2.6.1. Subjective Risk

**Figure 4:**
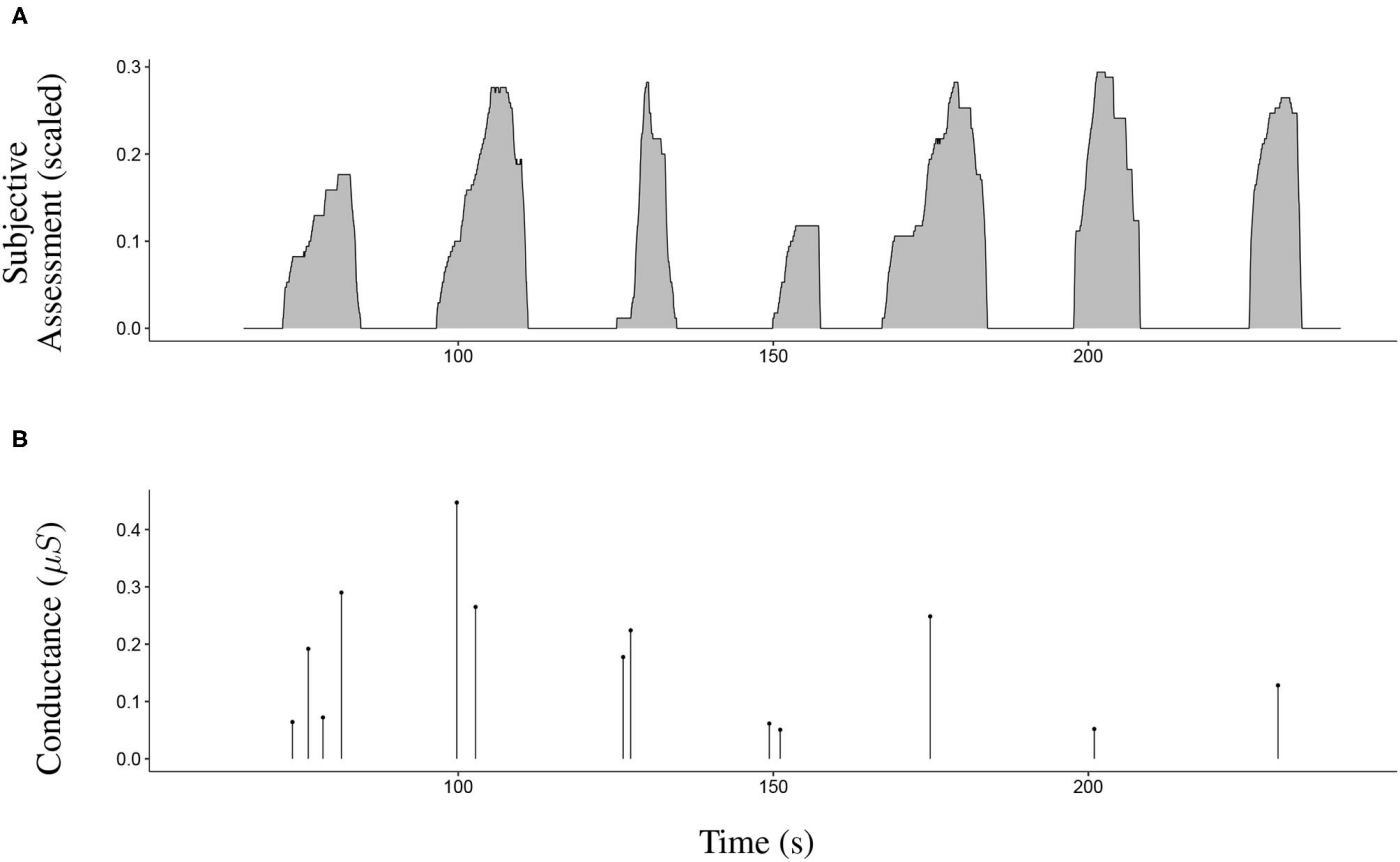
Sample of the collected data concerning seven avoidance manoeuvres. **(A)** The evolution of the subjective assessment during seven avoidance manoeuvres. The subjective assessment always returned to 0 between the manoeuvres. Indicator mSA corresponds to the maximum value of each manoeuvre. Indicator iSA corresponds to the area under each curve. **(B)** The skin conductance responses during the seven avoidance manoeuvres. Indicator mSCR corresponds to the maximum amplitude of the responses. Indicator nSCR corresponds to the number of skin conductance responses.

Two indicators were calculated to quantify the subjective risk assessment (denoted as “SA”) for each avoidance manoeuvre: The integrated risk assessed over time (*iSA*) and the maximum amplitude of the assessed risk (*mSA*). The indicator *iSA* can be considered a dynamic indicator as it accounts for both amplitude and temporality. It corresponds to the area under the curve for each maneuver filled with grey in Figure 4. The indicator *mSA* was calculated to provide a simple quantification of the participants' SA. It corresponds to the maximum value at each peak. As illustrated in Figure 4, the subjective assessment evolved differently between the manoeuvres and always returned to 0 between each pedestrian.

#### 2.6.2. Skin Conductance Response

The data were initially processed using the software program AcqKnowledge 5.0 (BIOPAC Systems, Inc., USA); MATLAB (MATLAB, 2018) and R (R Core Team, 2020) were then used to extract the indicators. Several manipulations had to be conducted to calculate the indicators. First, the raw data were pre-processed using AcqKnowledge according to the recommendations made by Braithwaite et al. (2013) and Findlay (2017). These included resampling at 78 Hz, moving median smoothing at 1 s and low-pass filtering at 1 Hz. The pre-processed data were then analyzed using the Ledalab application, specifically Version 3.4.8 from Benedek and Kaernbach (2010). This application is a MATLAB toolbox that was designed for isolating the tonic and phasic components of EDA. The method used was based on the following two steps:

1. The identification of the phasic component using Continuous Decomposition Analysis (CDA) with two parameters for the optimization of the deconvolution algorithm. This method, which is described in Benedek and Kaernbach's article (Benedek and Kaernbach, 2010), is based on a deconvolution algorithm to detect SCR. This technique is particularly effective for identifying and determining the characteristics of so-called “superimposed” responses (Boucsein, 2012).
2. The detection of SCR > 0.05 μS (the extraction of their onset and amplitude).

Finally, the indicators were calculated using the R software. For each avoidance manoeuvre, relative SCRs were selected only if their onset occurred no more than *w*_*start*_ seconds before the moment when the vehicle was next to a pedestrian and no more than *w*_*end*_ seconds after that moment. An avoidance manoeuvre could elicit an SCR only during the moment the participants started to perceive (assess) a collision risk. Therefore, the value *w*_*start*_ was computed for each participant, and two distinct moments for each manoeuvre were considered:

- The moment when the participant started to assess a non-zero value of collision risk;
- The moment when the vehicle was effectively level with a pedestrian.

This process resulted in 32 values (which corresponded to the total number of manoeuvres) per participant, which were averaged to find *w*_*start*_. The value of *w*_*end*_ was based on results from the literature (Boucsein, 2012; Droulers et al., 2013). SCR could be related to an avoidance manoeuvre only if it occurred in the 3 s following the moment when the vehicle was next to a pedestrian.

As for the subjective assessment data, two indicators were calculated to quantify EDA. These included the maximum amplitude of skin conductance responses (*mSCR*) and the number of skin conductance responses (*nSCR*). Figure 4 illustrates a sample of SCR data. The indicator *nSCR* corresponds to the number of SCRs during each manoeuvre, and *mSCR* corresponds to the maximum amplitude for all concerned SCRs.

## 3. Data Analysis

The analysis was based on the modeling of hybrid Bayesian (Denis and Scutari, 2015, Chapter 3) to study the relationship that may exist between subjective risk assessment indicators and the participants' skin conductance response indicators and the effects of the factors. Each Bayesian network proposed in this study includes four variables, which are also called “nodes.” A Bayesian network is represented by its directed acyclic graph (DAG), which graphically illustrates the relationships between its nodes. A node represents a variable that is associated with a statistical distribution whose parameters possibly depend on the other nodes. An arrow is used to specify that the distribution of a node depends on the value of another node. In this study, all directed acyclic graphs contain four nodes. Two nodes represent the independent factors TTC and offset. They were both assigned to discrete distributions whose probability mass functions were determined by the frequency of their values in the design of the experiment (cf. Table 1). Two other nodes, which were denoted “SA” and “SCR,” were respectively assigned to the subjective assessment and the skin conductance response indicators. Node SA (resp. SCR) designated either the *iSA* indicator or the *mSA* indicator (resp. *nSCR* and *mSCR* indicators).

**Table 1:** Value's frequencies for independent variables.

**Offset (m)**	**TTC (s)**				**Total frequency**
	**3.5**	**3**	**2.5**	**2**	
1.5	0.125	0.125	0.125	0.125	0.500
1	0.125	0.125	0	0	0.250
0.5	0.125	0.125	0	0	0.250
Total frequency	0.375	0.375	0.125	0.125	1.000

*This table illustrates the combinations of independent variables used in the experiment. The frequency for each value is given, and the totals for each row/column were added to provide details on the distribution of each variable*.

After an analysis of the distributions of the two indicators of subjective assessment was conducted, the original data was transformed to correct a positive skewness. A power of $\frac{1}{2}$ was applied to *mSA* values and a power of $\frac{1}{3}$ to *iSA* values. Moreover, to consider global distributions for all the participants, the transformed SA values were then centered and scaled by the participants. These transformations were performed to use Gaussian distributions for the node SA in the Bayesian networks. To ensure that this hypothesis on the distributions was relevant, a Shapiro-Wilk test was performed. The results are presented in Table 2. The transformations that were performed on the indicators resulted in more symmetrical distributions that can be assumed to be normal according to the statistics of the Shapiro-Wilk test (*p*>0.5 for both variables).

**Table 2:** Sample descriptive statistics and normality test of subjective assessment variables.

**Variable**	**Descriptive statistics**				**Shapiro Wilk test**	
	***n***	**M**	**SD**	**Skewness**	**W**	***p***
**Raw**						
mSA	640	0.401	0.252	0.653	0.951	0.000
iSA	640	1.548	1.426	1.431	0.865	0.000
**Transformed**						
${\left(\text{mSA}\right)}^{\frac{1}{2}}$	640	0.000	0.985	−0.010	0.999	0.974
${\left(\text{iSA}\right)}^{\frac{1}{3}}$	640	0.000	0.985	−0.016	0.998	0.701

*The raw variables correspond to the original data. The transformed data correspond to centered and scaled variables for each individual. These operations were performed after the power transformations of the initial values were made (that is $\frac{1}{2}$ for the indicator mSA and $\frac{1}{3}$ for the indicator iSA)*.

A preliminary analysis revealed that the SCR indicators had 46% of exactly zero. That means that only a part of an avoidance manoeuvre produced physiological responses. For this reason, a Tweedie compound Poisson distribution (Dunn and Smyth, 2005, 2008; Hasan and Dunn, 2012) was used for Node SCR in the Bayesian networks. This otherwise positive and continuous distribution has a positive mass at zero. The Tweedie compound Poisson distribution aided in estimating the distribution of the SCR indicators, as well as the probability of zero responses. To consider global distributions and homogenize the fluctuations between the data of each participant, indicators mSCR and nSCR were scaled per participant. As in the example provided by Dunn and Smyth (2005), an initial diagnostic (which has not been reported here) was performed to verify that the Tweedie approach of modeling the zeros and the positive observations together was adequate to estimate the parameters of the distribution.

For all node distributions, the parameters were estimated using the R software. More specifically, as in the method presented by Denis and Scutari (2015), the parameters of the factors TTC and offset were set as the actual frequency in the experiment (cf. Table 1), and the parameters of the Gaussian distribution were estimated by fitting linear models. The parameters of the compound Poisson distribution were estimated using the R package *cplm* (Zhang, 2013). Following the method presented by Denis and Scutari (2015), when a factor influenced a dependent node's (SA or SCR) distribution, the parameters were estimated for each value of the factor. For instance, eight parameters were estimated for the distribution of a Gaussian node that was influenced exclusively by the factor TTC (i.e., a mean and a standard deviation for each of the four levels of TTC). Concerning the graphs where both the factor TTC and offset influenced an indicator, a distribution was fitted for each of the eight combinations (cf. Table 1).

**Figure 5:**
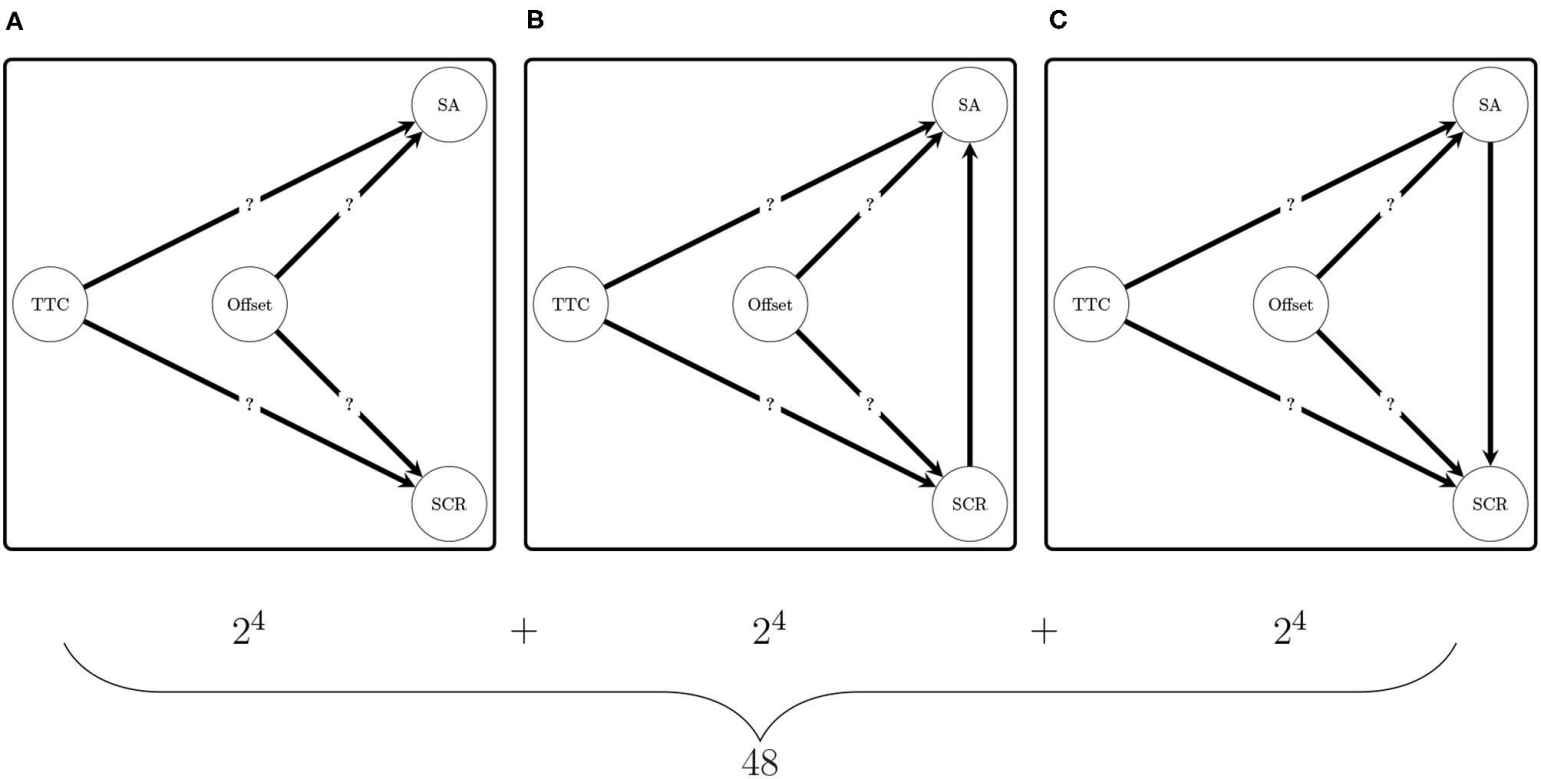
Directed acyclic graphs (DAG) compared in the experiment. **(A)** Illustrates the 16 DAGs that do not contain a relationship between SA and SCR nodes. **(B)** Illustrates the DAG that contains a relationship between the node SCR and the node SA. **(C)** Illustrates the DAG that contains a relationship between the node SA and the node SCR. The only DAG in **(A)** is consistent with the hypothesis of independence between the subjective assessment and the skin conductance response.

Forty-eight networks were computed regarding the four indicators mentioned previously (*iSA, mSA, nSCR*, and *mSCR*; see Figure 5). The consideration of four indicators rather than two (i.e., one for the SA and one for the SCR) allowed the amount of data that was used to analyze the effect of the factors and the relationship between the two risk perception types to be multiplied by four. To compare the Bayesian networks and select the more plausible one given the data, the Bayesian Information Criterion (BIC, Schwarz, 1978; Kass and Raftery, 1995; Raftery, 1995) was used. This is the criterion that is used for Bayesian network selection in the greedy search algorithm mentioned by Denis and Scutari (2015, p. 110). The procedure consists of favoring the network that has the lowest BIC score. For a Bayesian network, the Bayesian Information Criterion was calculated according to the following formula:


(1) BIC=−2ℒℒ+plog(n),

where ℒℒ is the joint log-likelihood of variables in the Bayesian network, *p* is the number of estimated parameters associated with the joint distribution of the variables in the network and *n* is the number of samples. The BIC allows non-nested Bayesian networks to be easily compared and is conservative regarding relationships (Raftery, 1999). That is, new relationships between nodes will only be significant concerning the BIC if they provide sufficient benefits regarding the overall likelihood. In the specific case of the Bayesian network, the BIC calculation was decomposed as the sum of the BIC at the four nodes (Denis and Scutari, 2015, p. 19):


(2) $$BIC=BIC^{\text{TTC}}+BIC^{\text{Offset}}+BIC^{\text{SA}}+BIC^{\text{SCR}},$$

where **BIC**^*x*^ is the value of the Bayesian information criterion computed for a node *x*. This equation results from the fact that the joint log-likelihood of a Bayesian network can itself be decomposed as the sum of the log-likelihood for each node when considering the relationships between them while estimating distribution parameters.

The grades of evidence from Kass and Raftery (Raftery, 1995, p. 139) were used to discuss the BIC differences in the values after the ranking process. Gaps larger than 2, 6, or 10 between two BIC values were considered positive, strong or very strong, respectively. Afterwards, the Hypothesis $H_{0}$, which states the independence between the subjective assessment and the skin conductance responses, requires that the best Bayesian networks (which were obtained for each combination of indicators) do not contain a relationship between the node SA and the node SCR. Conversely, it is sufficient for one of the best networks to contain a relationship between the node SA and the node SCR to reject Hypothesis $H_{0}$ in favor of $H_{1}$.

The best Bayesian networks were finally investigated to analyse the estimated distribution of each indicator. For indicators of subjective assessment, whose distributions were assumed to be Gaussian, the conditional mean estimates with confidence interval at 95% were represented. Additionally, when the influence of both factors appeared in the best Bayesian network, a cluster analysis was performed based on the Bayesian information criterion (Binder, 1978; Franzén, 2008). We considered all the conditions resulting from the interaction of the TTC and offset factors, that is, eight possible levels. The objective was then to find out if these eight levels gave rise to different distributions of the indicator considered (*mSA* or *iSA*) or if they could be grouped into a smaller number. For this purpose, models were built for all possible groupings, that is, 4140 possible partitions in accordance with the eighth Bell number (Rota, 1964). The R package *partitions* was used for this (Hankin, 2006; Hankin and West, 2007). Then the BIC value was calculated for all the models. The best model was selected by using the Raftery's grades of evidence (Raftery, 1995, p. 139).

For indicators of SCR, which were assumed to follow Tweedie distributions, conditional mean estimates were represented, as well as the probability of zeros. These two representations were used to provide a more complete preview of the functioning of an effect given this specific Tweedie distribution. Both representations were useful for providing a more complete picture of how an effect worked given this Tweedie distribution. Since SCRs were quite rare in the data, it was interesting to visualize the evolution of the mean of an indicator in parallel with the probability of no response.

## 4. Results

### 4.1. The Relationship Between the Two Types of Risk Perception Measures

All the possible Bayesian networks were compared to address the two theoretical hypotheses: $H_{0}$, which states that the two types of risk perception are independent, and $H_{1}$, which states that a relationship exists between the two types of risk perception. The best Bayesian networks were selected for each of the four combinations of indicators according to the BIC. Table 3 presents the three best Bayesian networks per combination regarding their value of BIC. In this table, the structure of the relationships between the Bayesian networks was represented by the likelihood decomposition for node SA and node SCR. This notation was adopted to succinctly reflect the dependence between the distributions of subjective assessment measures and skin conductance responses given the factors TTC and offset.

**Table 3:** Values of the BIC of the three best models by indicator combination.

**Likelihood decomposition**		**BIC**
${ℒ}_{\left(\text{mSA, mSCR}|\text{TTC, Offset}\right)}$		
${ℒ}_{\left(\text{mSA}|\text{TTC}\right)}$	× ${ℒ}_{\left(\text{mSCR}|\text{TTC}\right)}$	6044.724*
${ℒ}_{\left(\text{mSA}|\text{TTC, Offset}\right)}$	× ${ℒ}_{\left(\text{mSCR}|\text{TTC}\right)}$	6045.361*
${ℒ}_{\left(\text{mSA}|\text{TTC, mSCR}\right)}$	× ${ℒ}_{\left(\text{mSCR}|\text{TTC}\right)}$	6050.928
${ℒ}_{\left(\text{mSA, nSCR}|\text{TTC, Offset}\right)}$		
${ℒ}_{\left(\text{mSA}|\text{TTC}\right)}$	× ${ℒ}_{\left(\text{nSCR}|\text{mSA}\right)}$	5979.376*
${ℒ}_{\left(\text{mSA}|\text{TTC, Offset}\right)}$	× ${ℒ}_{\left(\text{nSCR}|\text{mSA}\right)}$	5980.013*
${ℒ}_{\left(\text{mSA}|\text{TTC}\right)}$	× ${ℒ}_{\left(\text{nSCR}|\text{TTC}\right)}$	6009.594
${ℒ}_{\left(\text{iSA, mSCR}|\text{TTC, Offset}\right)}$		
${ℒ}_{\left(\text{iSA}|\text{TTC, Offset}\right)}$	× ${ℒ}_{\left(\text{mSCR}|\text{TTC}\right)}$	6038.815*
${ℒ}_{\left(\text{iSA}|\text{TTC}\right)}$	× ${ℒ}_{\left(\text{mSCR}|\text{TTC}\right)}$	6053.426
${ℒ}_{\left(\text{iSA}|\text{TTC, Offset}\right)}$	× ${ℒ}_{\left(\text{mSCR}|\text{TTC,iSA}\right)}$	6057.045
${ℒ}_{\left(\text{iSA, nSCR}|\text{TTC, Offset}\right)}$		
${ℒ}_{\left(\text{iSA}|\text{TTC, Offset}\right)}$	× ${ℒ}_{\left(\text{nSCR}|\text{iSA}\right)}$	5983.842*
${ℒ}_{\left(\text{iSA}|\text{TTC}\right)}$	× ${ℒ}_{\left(\text{nSCR}|\text{iSA}\right)}$	5998.453
${ℒ}_{\left(\text{iSA}|\text{TTC, Offset}\right)}$	× ${ℒ}_{\left(\text{nSCR}|\text{TTC}\right)}$	6003.685

*For the sake of simplicity, the likelihood (denoted ℒ) decompositions are only noted for nodes SA and SCR. However, the values in the BIC column correspond with the total BIC of the Bayesian network. The distribution parameters have not been specified here. The indicators of subjective assessment were assumed to follow a Gaussian distribution. The indicators of skin conductance response were assumed to follow a Tweedie distribution. The asterisks indicate the best Bayesian networks according to Raftery's degree of evidence*.

Table 3 reveals that one unique Bayesian network was selected for the indicator *iSA*. The results revealed that this indicator was influenced by the combination between levels of TTC and offset. Concerning the indicator *mSA*, the best Bayesian network (i.e., the Bayesian network with the lowest BIC score) could not be definitely distinguished from the Bayesian networks ranked in second position. The BIC difference between those two best Bayesian networks was not significant regarding the Raftery's grade of evidence (Raftery, 1995). Essentially, the difference was lower than 2. The dependence structure of those two best Bayesian networks were similar except for the dependence between the indicator *mSA* and the factor offset, which appeared only in the Bayesian network that was ranked in second position. This result means that there is not enough evidence to conclude with certainty that the factor offset influenced the indicator *mSA*.

**Figure 6:**
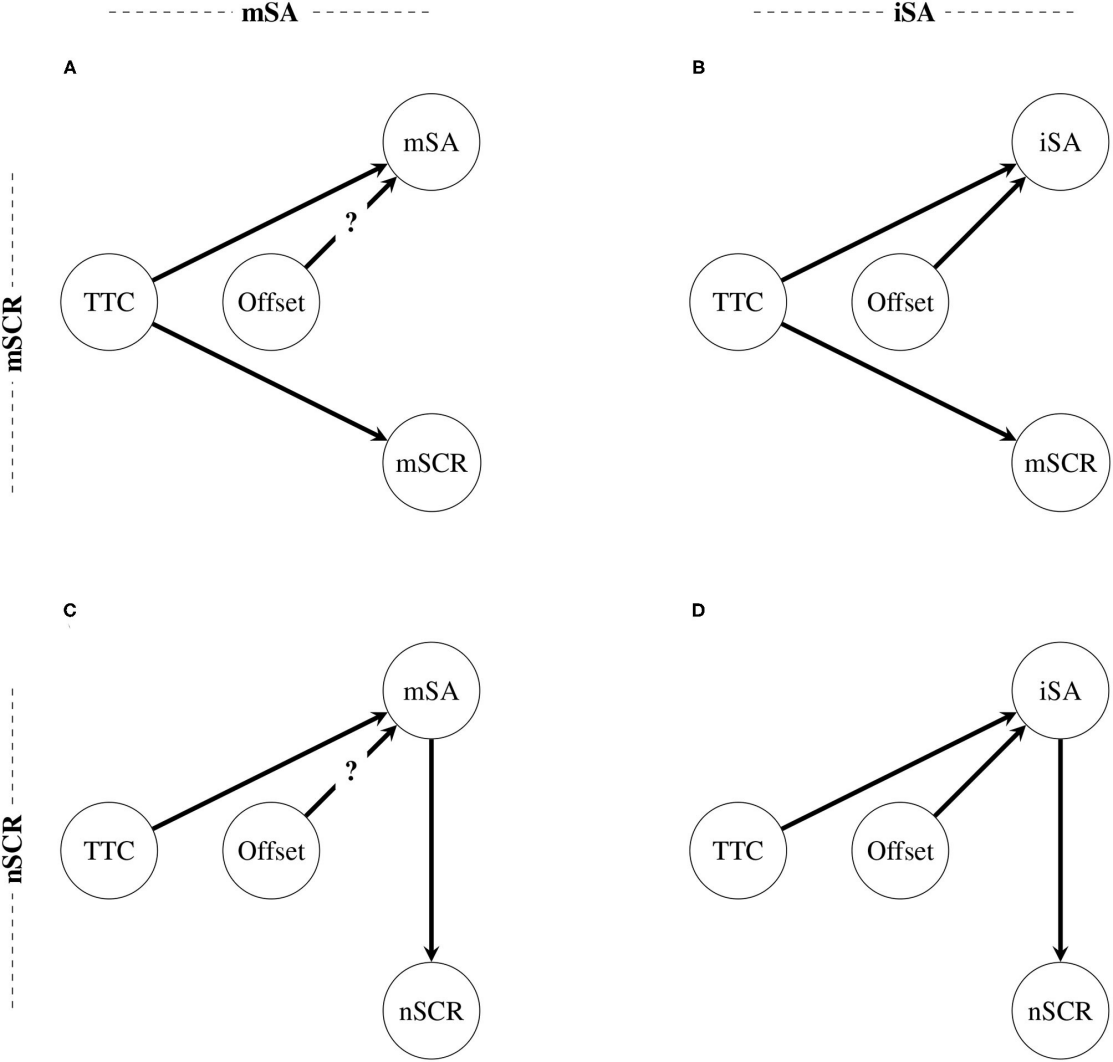
Directed acyclic graphs of the best Bayesian networks by indicators combination. **(A)** The relationships between the factors and the indicators *mSA* and *mSCR*. **(B)** The relationships between the factors and the indicators *iSA* and *mSCR*. **(C)** The relationships between the factors and the indicators *mSA* and *nSCR*. **(D)** The relationships between the factors and the indicators *iSA* and *nSCR*. A question mark was introduced to the illustration of relationship between node Offset and node *mSA* because the degree of evidence for this relationship is not sufficient regarding the BIC given the thresholds developed by Raftery (1995), which were adopted in this study.

**Figure A1:**
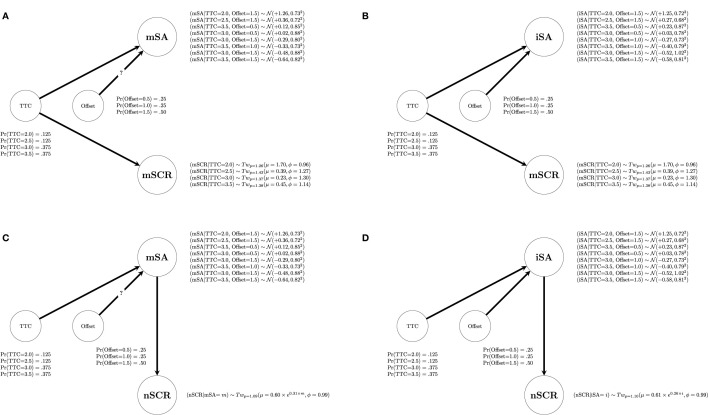

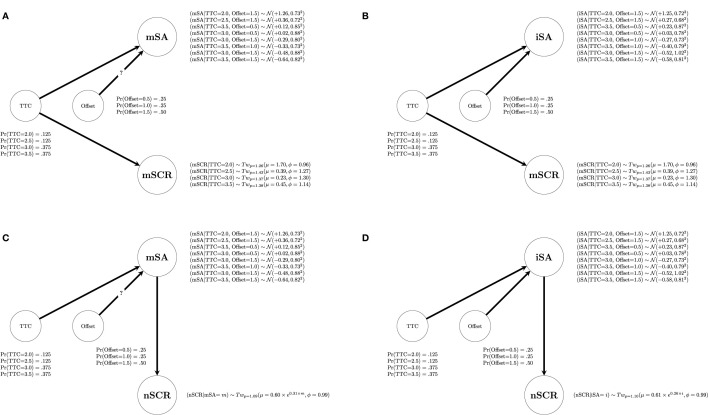
The four best directed acyclic graphs with detailed distributions. **(A)** The relationships between the factors and the indicators mSA and mSCR. **(B)** The relationships between the factors and the indicators iSA and mSCR. **(C)** The relationships between the factors and the indicators mSA and nSCR. **(D)** The relationships between the factors and the indicators iSA and nSCR. The question mark on the relationship between node Offset and node mSA indicates that the degree of evidence for this relationship is not sufficient according to the BIC thresholds developed by Raftery (1995).

The directed acyclic graphs of the best Bayesian networks revealed by Table 3 are illustrated in Figure 6. Details about the estimated coefficients for each distribution are provided in Figure A1. Since there was not enough evidence to conclude the relationship between the factor offset and the indicator *mSA*, a question mark was placed on the arrows between these two nodes. Figure 6 illustrates two results.

- First, the subjective risk assessment depends on the two factors: Indicator *mSA* definitely depends on the factor TTC and possibly on the factor offset (see Figures 6A,C), and indicator *iSA* depends on both factors TTC and offset (see Figures 6B,D).
- Secondly, whereas the maximum amplitude of the skin conductance responses (indicator *mSCR*) depends only on the TTC (see Figures 6A,B), the number of skin conductance responses depends only on the subjective risk assessment (see Figures 6C,D). This result permitted the rejection of $H_{0}$ in favor of $H_{1}$.

Consequently, the results support the existence of a relationship between the two types of risk perception.

### 4.2. The Analysis of Risk Perception Variations According to the Factors

**Figure 7:**
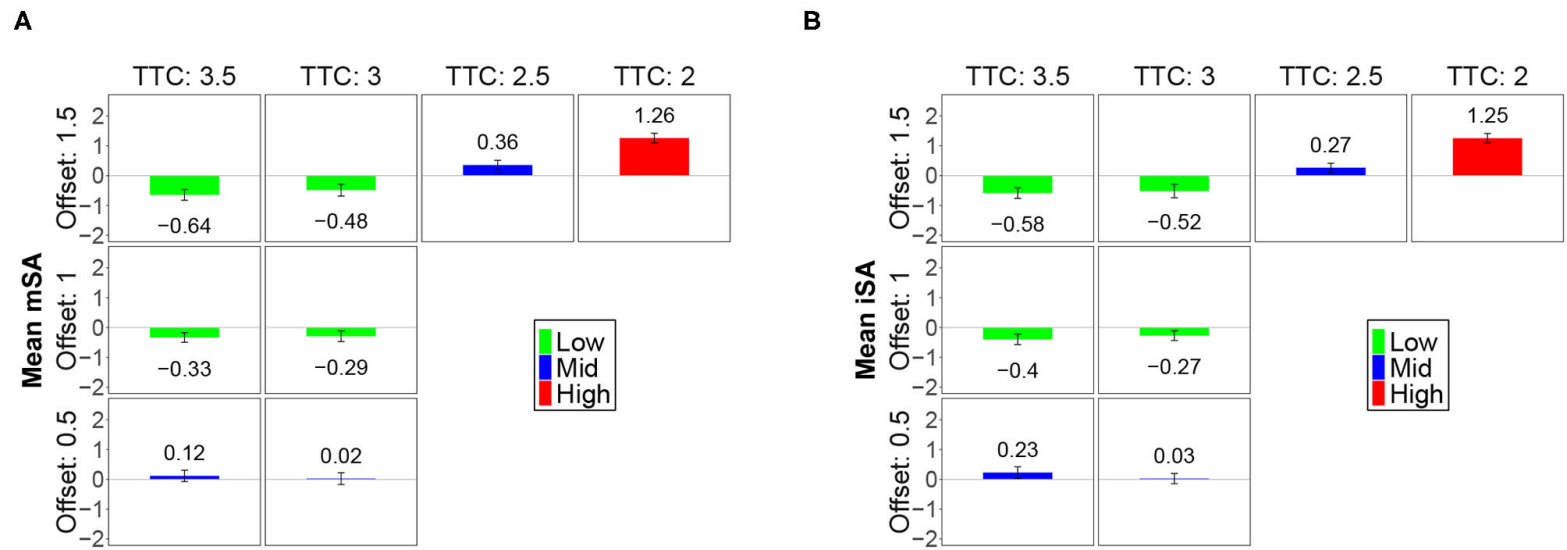
Means for the SA variables for level combinations of TTC and offset. **(A)** The means of the indicator *mSA* in combination with the TTC and offset. **(B)** The means of the indicator *iSA* in combination with the TTC and offset. To better distinguish differences, confidence intervals at 95% are represented for each mean.

A coefficient analysis was performed for the subjective risk assessment obtained for each observed combination of the factors. Figure 7 presents the means of the subjective risk assessment obtained for the indicators *mSA* (Figure 7A) and *iSA* (Figure 7B). The means were typically represented by bars that were proportional to their value, and 95% confidence intervals were plotted to better visualize significant differences. An inspection of this figure reveals that the patterns of the results (as a function of the combinations of factor) were similar for both indicators. A cluster analysis was performed to better identify the similarities and differences between the means of a given indicator. This procedure relied on the Bayesian information criterion to find the optimal groupings of factor level combinations based on the data. It resulted in three homogeneous categories that were characterized as functions of the effect on risk perception: Low, mid, and high. These three categories have been detailed in the following manner:

- When the values of the factors TTC and offset were both high (superior or equal to 3.0 s for the TTC and superior or equal to 1.0 m for the offset), the risk was perceived to be low; the means of the subjective assessment indicators were lower than the average.
- When the level of the TTC was median (2.5 s) or when the offset was small (0.5 m), the risk was perceived to be moderate; the means of the subjective assessment indicators were close to the average.
- When the level of the TTC was small (2.0 s), the risk was perceived to be high; the means of the subjective assessment indicators were significantly greater than the average.

**Figure 8:**
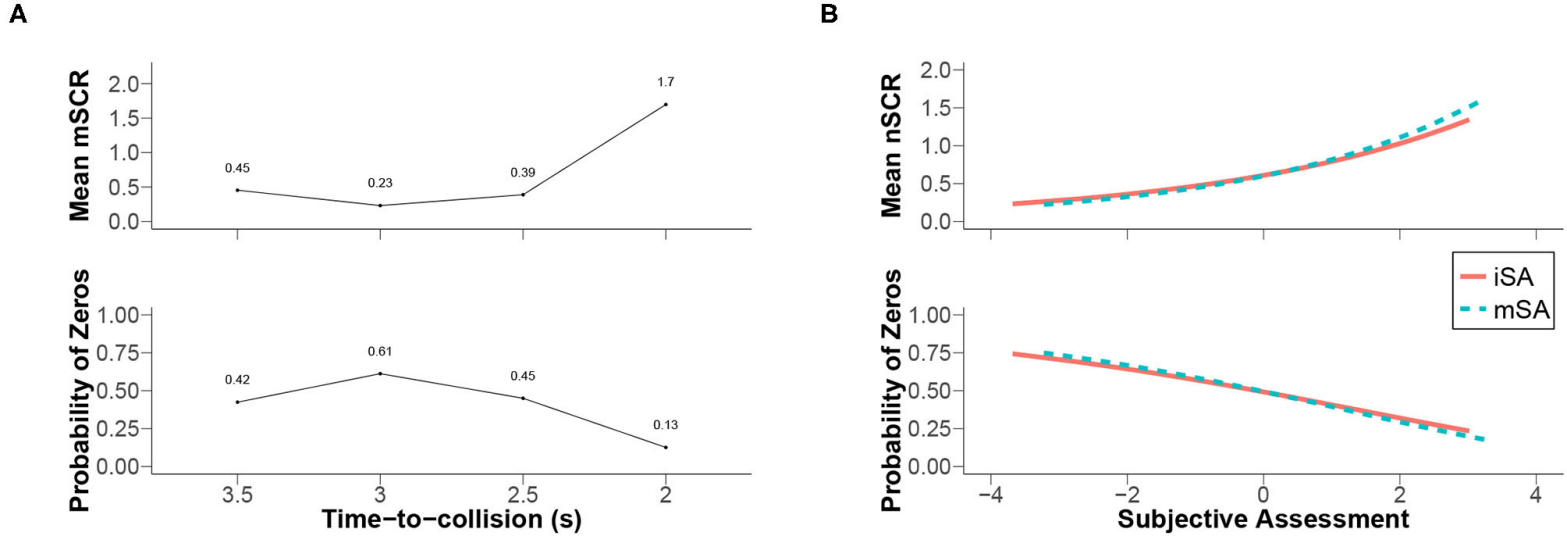
Conditional means of SCR indicators according to the best Bayesian networks. **(A)** The means of the indicator *mSCR* in combination with the TTC values. **(B)** The means of the indicator *nSCR* as a function of the SA values. The probability of zeros was derived from the estimated parameters of each distribution (Tweedie). The relationships between the parameters of such distributions were, for instance, presented by Zhang (2013, eq. 2.2).

Figure 8A presents the estimated means of the indicator *mSCR* and the estimated probabilities of zeros according to the levels of factor TTC. The examination of this figure revealed that the probability of zeros (i.e., of not observing skin conductance responses in the participants) decreased as the mean of the indicator *mSCR* increased. Moreover, the maximum amplitude was obtained when the level of the TTC was small (2.0 s). Consequently, the later the vehicle initiated its avoidance manoeuvre, the greater the chance of observing skin conductance responses in the participants became. Furthermore, it can be noted that the lowest level of the TTC produced a similar impact on the two risk perception systems. In this particular case, the risk was perceived to be high based on the indicators of the two types of risk perception.

The analysis of the directed acyclic graphs of the best Bayesian networks (cf. Figure 6) revealed that the number of skin conductance responses depended on the subjective risk assessment indicators (i.e., *mSA* and *iSA*) rather than on the TTC and offset levels. This result was considered an example of the relationship between the two types of risk perception as it refuted their independence. Figure 8B illustrates this relationship (using estimated parameters of the Tweedie distribution assumed for the indicator *nSCR* detailed in Figure A1). The mean of the number of skin conductance responses was represented as a function of the subjective risk assessment indicators. The mean of the number of skin conductance responses varied similarly for indicators *mSA* and *iSA*. Per the statistical model used to fit the distribution, the link between the number of skin conductance responses and the indicators of subjective risk assessment was exponential. The higher the subjective assessment indicators were, the higher the number of skin conductance responses were. Additionally, the probability of having no skin conductance response (i.e., the probability of zeros) was high when the indicators of subjective risk assessment were low. The probability of not observing a skin conductance response in the participants decreased as the subjective assessment indicators decreased.

## 5. Discussion

This study sought to characterize the perception of risk made by a passenger in an autonomous vehicle that was moving in a space shared with pedestrians. For this purpose, the subjective risk assessment and the skin conductance responses were collected in parallel to better understand how the two perception systems (“risk as feeling” and “risk as analysis”) act in such a situation.

The result of the Bayesian network modeling revealed that the hypothesis concerning independence between the two risk perception systems must be rejected under the TTC and offset conditions that the study evaluated. Although the maximum amplitude of the skin conductance responses is impacted by small TTC values, the analysis demonstrated that the number of skin conductance responses depends only on the subjective risk assessment. These results, therefore, support the hypothesis that claims that the two risk perception systems are not completely interdependent as they may influence one another independently of environmental factors (Loewenstein et al., 2001; Slovic et al., 2004). Nevertheless, since the subjective risk assessment was more sensitive to external conditions than the skin conductance responses, it is more likely that the subjective risk can induce skin conductance responses than the opposite. This conclusion will have to be confirmed by further studies.

The results revealed that there are three classes of situations. When TTC and offset were simultaneously high, between 3.0 and 3.5 s and between 1.0 and 1.5 m respectively, the risk was perceived as low. When the TTC was intermediate (2.5 s) or when the offset was low (0.5 m), the risk was perceived as moderate. Finally, when the TTC was small (2.0 s), the perceived risk was higher than in all other situations. Thus, the results confirmed that the TTC strongly determines the perception of a collision risk during an avoidance manoeuvre (Lee, 1976; Bootsma and Craig, 2003). The 2.5 s threshold appears to be consistent with the recommendations that were made by the U.S. Department of Transportation (NHTSA, 2013). Indeed, the minimum warning thresholds recommended in the test protocols for collision warning systems are 2.1, 2.4, and 2.0 s when the vehicle respectively approaches a fixed, decelerating or low-speed obstacle. During an avoidance manoeuvre, when the vehicle passed a pedestrian, the lateral offset also influenced risk perception. The closer the vehicle was to the pedestrian, the greater the subjective risk became. However, the results demonstrate that the offset had a smaller effect on the SA than the TTC did and may not have affected the SCR. Hence, subjective risk perception has evolved in the same way as EDA on average but not necessarily with the same magnitude.

The subjective assessment of collision risk is influenced by vehicle dynamics. The non-linearity of the effects observed on the indicators reveals that risk perception does not result from the simple relationship between the probability of a hazard and its importance. Rather, the results evoke a threshold effect as suggested by Boer (2006). Each passenger built up safety margins and would only perceive a risk when the vehicle approached a pedestrian and violated these margins. The passenger's risk perception would, therefore, result from a continuous confrontation between the vehicle's trajectory and their safety margins. These findings are compatible with the concept of the “safe field of travel” that was introduced by Gibson and Crooks (1938), according to which an individual represents a dynamic area in which their vehicle can navigate safely. In comparison with the experiment conducted by Ferrier-Barbut et al. (2018), who used a virtual-reality helmet to test the impact of proximity between pedestrians and a vehicle, this experiment utilized a driving simulator that lacked a physical vehicle cab, which may have made it difficult to estimate the vehicle's width and its lateral distance from object (Mecheri and Lobjois, 2018). Although Walker et al. (2019b) demonstrated that medium-level driving simulators remain appropriate for the study of risk perception, the lack of a physical cab simulator and the absence of real danger may have limited the participants' abilities to gauge their proximity to the pedestrians.

The participants' physiological responses to approaching pedestrians reflect the activation of the sympathetic nervous system that operates parallel to subjective evaluation. As Choi et al. (2019) stated, the sympathetic nervous system can only react to a certain level of danger, and this can cause variations in certain physiological variables. The analysis of the EDA in this experiment confirmed this idea by revealing an increase in indicators mostly during high subjective risk assessment.

This study investigated how passengers perceive risk in autonomous vehicles that are navigating areas that include pedestrians. The aim was to better understand the fundamental mechanisms of risk perception in the particular case of shared spaces. Understanding how vehicle-environment dynamics influence the perception of a vehicle's passengers and pedestrians could help researchers create and implement motion algorithms that are compatible with the safety margins of all agents in a system design approach. This could also condition the acceptability of autonomous vehicles. This study presents preliminary results regarding this topic. However, many other parameters of vehicle-environment dynamics must be studied to progress. The question regarding the variables that were chosen to evaluate the passenger's feelings based on subjective evaluations or physiological measurements is essential. These results demonstrate that the measurements of the two types of indicators are not independent but are instead complementary.

## 6. Conclusion

This study highlighted the relevance of declarative and physiological measures of the real-time analysis of risks perceived by those in an autonomous vehicle. The results obtained are consistent with the literature concerning the effects of the manipulated variables. The value of the TTC at the beginning of a pedestrian avoidance manoeuvre and the lateral distance left between the vehicle and the pedestrian do affect the subjective risk. This study has demonstrated that physiological and subjective indicators are not independent but do not always lead to the same results, which supports the proposition made by Février et al. (2011). They stated that declarative and physiological measures are not redundant but complementary. This experience has demonstrated that one must be careful to not make a universal conclusion based on a single indicator in studies on risk perception. Subjective evaluations (risk as analysis) may be more sensitive to low-risk situations than physiological responses (risk as feeling) in particular. This work and its conclusions would benefit from being replicated in more realistic and complex environments. The safety margins that were tested in the driving simulator may not fully match those tolerated in a real autonomous vehicle. In a simulator or vehicle, the relationship between the two risk perception systems could be evaluated in a more complex and realistically modeled space, for example by varying pedestrian behaviors. As the environment becomes more complex, new risk perception factors (in addition to vehicle-environment dynamics) could be revealed. For example, passenger perception could be affected by unpredictability or lack of understanding of the autonomous vehicle state. The modeling approach we adopted could also be implemented to assess differences in risk perception between active drivers and passengers. Indeed, Basu et al. (2017) have shown that passengers prefer a more defensive driving style than when they are themselves in control of the vehicle. Finally, other works could be interested in the interaction modalities to be considered for the communication of information to the passenger-drivers so that they feel more secure (Bengler et al., 2020).

## Data Availability Statement

The dataset and the algorithm used in this study are available as a R package that can be downloaded from: https://gitlab.univ-nantes.fr/petit-j-2/bnscore.

## Ethics Statement

The studies involving human participants were reviewed and approved by the non-interventional research ethics committee of Nantes University (CERNI, num. 10032021). The patients/participants provided their written informed consent to participate in this study.

## Author Contributions

JP, CC, and FM designed the study, contributed to the data modeling and statistical analysis, and wrote the manuscript. JP conducted the experiment. All authors contributed to the article and approved the submitted version.

## Funding

This study was supported by the French National Research Agency (Agence Nationale de la Recherche, HIANIC project, Grant no. ANR-17-CE22-0010). The authors thank the ELICC Doctoral School (n°603) for its contribution to the linguistic correction of the article.

## Conflict of Interest

The authors declare that the research was conducted in the absence of any commercial or financial relationships that could be construed as a potential conflict of interest.

## Publisher's Note

All claims expressed in this article are solely those of the authors and do not necessarily represent those of their affiliated organizations, or those of the publisher, the editors and the reviewers. Any product that may be evaluated in this article, or claim that may be made by its manufacturer, is not guaranteed or endorsed by the publisher.
